# *Arcanobacterium haemolyticum* Phospholipase D Enzymatic Activity Promotes the Hemolytic Activity of the Cholesterol-Dependent Cytolysin Arcanolysin

**DOI:** 10.3390/toxins10060213

**Published:** 2018-05-23

**Authors:** Patrick S. Gellings, David J. McGee

**Affiliations:** Department of Microbiology and Immunology, Louisiana State University Health Sciences Center-Shreveport, 1501 Kings Highway, P.O. Box 33932, Shreveport, LA 71130-3932, USA; pgelli@lsuhsc.edu

**Keywords:** sphingomyelinase, cholesterol-dependent cytolysin, *Arcanobacterium haemolyticum*, enzyme mutation, protein purification

## Abstract

Arcanolysin, produced by the human pathogen *Arcanobacterium haemolyticum*, is a cholesterol-dependent cytolysin. To mediate the pore-formation process, arcanolysin is secreted by *A. haemolyticum* and then must interact with cholesterol embedded within a host membrane. However, arcanolysin must compete with membrane components, such as the phospholipid sphingomyelin, to interact with cholesterol and form pores. Cholesterol forms transient hydrogen bonds with the extracellular portion of sphingomyelin, shielding cholesterol from extracellular factors, including arcanolysin. *A. haemolyticum* also produces a sphingomyelin-specific phospholipase D, which removes the choline head from sphingomyelin, leaving cyclic-ceramide phosphate and eliminating the potential for cholesterol sequestration. We hypothesized that the enzymatic activity of phospholipase D decreases sphingomyelin-mediated cholesterol sequestration and increases cholesterol accessibility for arcanolysin. Using purified arcanolysin and phospholipase D, we demonstrate that the enzymatic activity of phospholipase D is necessary to promote arcanolysin-mediated hemolysis in both time- and concentration-dependent manners. Phospholipase D promotion of arcanolysin-mediated cytotoxicity was confirmed in Detroit 562 epithelial cells. Furthermore, we determined that incubating phospholipase D with erythrocytes corresponds with an increase in the amount of arcanolysin bound to host membranes. This observation suggests that phospholipase D promotes arcanolysin-mediated cytotoxicity by increasing the ability of arcanolysin to bind to a host membrane.

## 1. Introduction

*Arcanobacterium haemolyticum* is a Gram-positive bacterium that is a causative agent of pharyngitis and wound infections in humans [1,2,3,4]. Little is known regarding the lifecycle and pathogenesis of *A. haemolyticum* despite its designation as an emerging pathogen by the Centers for Disease Control and Prevention over 20 years ago [1]. *A. haemolyticum* predominately infects adolescents and is the causative agent in approximately 240,000–480,000 cases of pharyngitis worldwide each year [1,2,3,4]. To date, only two *A. haemolyticum* putative virulence factors have been partially characterized: Arcanolysin (ALN) and phospholipase D (PLD). 

ALN is a member of the pore-forming cholesterol-dependent cytolysin (CDC) family [5,6]. Over 50 different CDCs have been identified, predominately from Gram-positive bacteria [6]. The steps involved in the CDC pore-formation process are well understood: monomer binding, oligomerization, β-sheet formation and insertion for final pore formation and membrane disruption [7]. As their name implies, nearly all members of the CDC-family, including ALN, rely on interactions with membrane cholesterol to mediate the pore formation process [5,7]. Apart from the knowledge that ALN has considerable specificity for human erythrocytes over rabbit, ovine or bovine cells and a dependence on membrane cholesterol for pore-formation, very little is known regarding neither the biochemistry of ALN nor its role in *A. haemolyticum* pathogenesis [5]. 

The other *A. haemolyticum* virulence factor, PLD, is understood to be universally produced by all *A. haemolyticum* isolates [8]. Bacterial phospholipase D enzymes commonly hydrolyze both sphingomyelin (SM) and phosphatidylcholine (PC) and contain a His-X-Lys-X4-Asp (HKD) motif within the catalytic site [9]. *A. haemolyticum* PLD, however, does not contain the traditional HKD motif and instead causes the unique cyclization of SM or lysophosphatidylcholine (LPC), while having minimal effect on PC [10]. Interestingly, *A. haemolyticum* PLD is most similar to a series of *Corynebacterium spp*. phospholipase D enzymes as well as a multitude of fungal pathogens, most of which have received little to no interest from the scientific community. As a result of its unique properties, *A. haemolyticum* PLD converts SM or LPC into choline and either cyclic-ceramide phosphate (CCP) or cyclic-phosphatidic acid (CPA), respectively [10]. The ramifications of its unique biochemistry have not been fully investigated, but preliminary studies suggests PLD may impact bacterial adherence and invasion, host plasma membrane lipid raft formation and host cell necrosis [8].

Various membrane lipids are known to interact with one another within a host membrane. One such example exists between cholesterol and SM. Cholesterol molecules are embedded within a membrane but display a hydroxyl group to the outside environment [11]. Similar to cholesterol, the majority of a SM molecule is membrane-embedded but presents a hydrophilic choline head group to the extracellular environment [11]. The hydroxyl group of cholesterol and a portion of the hydrophilic head of SM form a transient hydrogen bond, creating an “umbrella-like” covering over cholesterol [11]. This SM covering shields cholesterol from extracellular factors, including ALN [11]. Removal of the choline head of SM eliminates the possibility of cholesterol-SM interactions, thus increasing the pool of accessible cholesterol [12]. Based on the cholesterol-dependence of ALN, the substrate specificity of PLD, and the cholesterol-SM interaction, we hypothesized that the two *A. haemolyticum* virulence factors may work in tandem to promote ALN pore-formation.

Using purified PLD or ALN and human erythrocytes, we demonstrate that the enzymatic activity of PLD promotes ALN-mediated hemolysis in both time- and concentration-dependent manners. Further, we observed an increase in ALN-membrane binding as a result of incubating erythrocytes with PLD. Overall, this study demonstrates a previously unidentified synergistic relationship among the two known virulence factors of *A. haemolyticum*: ALN and PLD. 

## 2. Results

### 2.1. A. haemolyticum Produces at Least Two Products Which Contribute to Hemolytic Activity of Bacterial Supernatants

A diagnostic hallmark of *A. haemolyticum* is its strong β-hemolysis when grown on agar plates supplemented with human blood [1]. The size of the hemolytic rings suggests that one or more factors secreted by *A. haemolyticum* possess hemolytic activity.

**Figure 1:**
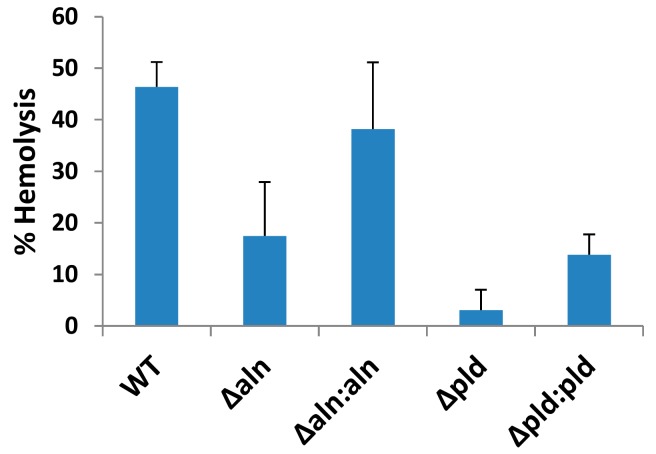
Absence of PLD or ALN decreases *A. haemolyticum* hemolysis levels. Overnight cultures of ATCC 9345 *A. haemolyticum*, Δ*aln*, Δ*pld* or their complements were centrifuged and culture supernatants were added to 2% human erythrocytes for 30 min at 37 °C. Samples were then centrifuged and the OD_415_ of the supernatants were measured and used to calculate the % hemolysis of each treatment. Results are representative of at least three independent experiments conducted in triplicate with error bars representing standard deviation of the mean.

Analysis of the *A. haemolyticum* genome suggests the presence of multiple, putative virulence factors, among which includes two factors that specifically target host membrane integrity: a sphingomyelin-specific phospholipase D (PLD) [8,10] and the CDC named ALN [5]. To measure the effect ALN and PLD have on the hemolytic capabilities of *A. haemolyticum*, supernatants were isolated from WT, Δ*pld*, Δ*aln*, and the corresponding complemented strains of *A. haemolyticum* and hemolytic activity was assessed (Figure 1). WT *A. haemolyticum* supernatants lysed 46.3% of erythrocytes in the assay while supernatants from the Δ*aln* and Δ*pld* strains lysed 17.5% and 3.1% of the erythrocytes, respectively (Figure 1). Complementing either mutant strain restores hemolysis, though not to the level of WT *A. haemolyticum* (Figure 1) which may be due to issues such as titration of limited regulatory factors or the fact that mutants were rescued with a multi-copy plasmid. These data suggest that both ALN and PLD contribute to hemolysis by the bacterium. Attempts to construct a Δ*pld*Δ*aln* double mutant have so far been unsuccessful. 

### 2.2. Purified His-ALN or His-PLD Is Sufficient to Cause Hemolysis of Human Erythrocytes

**Figure 2:**
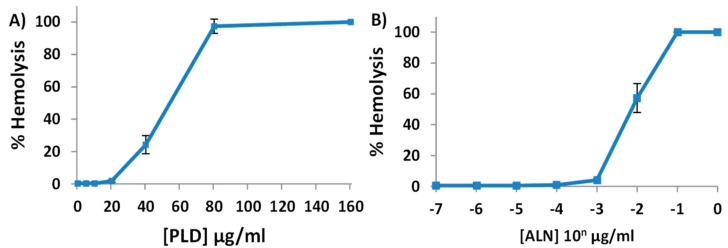
Purified PLD or ALN from *A. haemolyticum* is sufficient to induce hemolysis of human erythrocytes. Various concentrations of purified His-PLD (**A**) or His-ALN (**B**) were suspended in 1× PBS and added to 2% human erythrocytes for 30 min at 37 °C. % hemolysis was then determined as outlined in Figure 1. Results are representative of three independent experiments conducted in triplicate with error bars representing standard deviation.

When purified, CDCs, including ALN, are potent hemolysins [5,6,7]. However, it was previously reported that purified *A. haemolyticum* PLD contains no innate hemolytic activity by itself [8]. The observation that PLD does not contain hemolytic activity seemingly contradicts our data (Figure 1), which suggests PLD contributes to the hemolytic activity of *A. haemolyticum* supernatants. This apparent contradiction might be due to insufficient concentrations of purified PLD in prior experiments or its requirement of an unknown cofactor. To verify the hemolytic activity of PLD and ALN, His_6_ tagged recombinant ALN and PLD (His-ALN and His-PLD) were purified and the hemolytic activities of the individual proteins were measured against human erythrocytes. After 30 min incubation, sufficient concentrations of either His-ALN or His-PLD were determined to induce hemolysis of human erythrocytes, though the minimum concentration of His-ALN (20 ng/mL) was approximately 1000-fold lower than the minimum concentration of His-PLD necessary to cause hemolysis (20 µg/mL) (Figure 2).

As a negative control, *H. pylori* RocF was similarly purified and subject to a similar concentration-dependent hemolysis assay [13]. Regardless of the concentration of RocF used, no hemolysis was observed (data not shown). Thus, either ALN or PLD are sufficient to cause hemolysis individually as purified proteins and may contribute to the hemolytic activity of *A. haemolyticum* supernatants. 

### 2.3. A. haemolyticum PLD Promotes ALN-Mediated Hemolysis in Both Time- and Concentration-Dependent Manners

**Figure 3:**
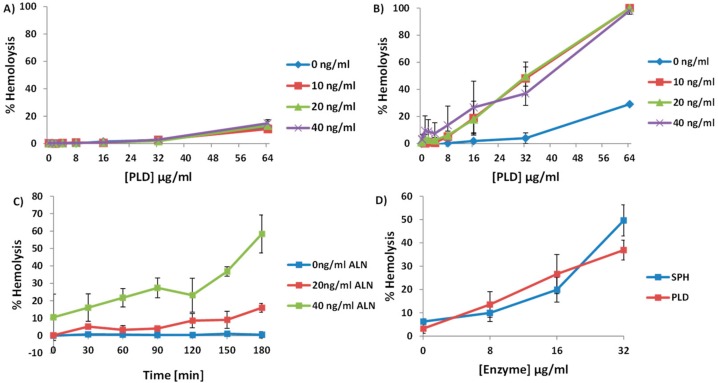
Pre-incubating human RBCs with PLD promotes ALN-mediated hemolysis in time- and concentration-dependent manners. (**A**) 0–40 ng/mL ALN was incubated with 4% human erythrocytes for 30 min at room temperature, followed by the addition of 0–64 µg/mL of PLD for an additional 30 min, but little to no detectable hemolysis was observed. (**B**) When the reciprocal experiment was performed, erythrocyte incubation with PLD followed by the addition of ALN, there was a significant increase in hemolysis. (**C**) Equal parts 4% erythrocytes and 4 µg/mL PLD were incubated at room temperature for between 0 and 180 min. At the end of each time point, equal parts PLD-treated erythrocytes and either 0, 20, or 40 ng/mL ALN were incubated together for an additional 30 min. Each experiment was conducted three times. (**D**) Purified SPH or PLD were incubated with erythrocytes in a similar manner as previously mentioned, followed by the addition of 40 ng/mL ALN. Both SPH and PLD were able to promote ALN-mediated hemolysis in a similar manner. Results are representative of at least three independent experiments conducted in triplicate with error bars representing standard deviation of the mean.

It was previously established that *A. haemolyticum* PLD contains sphingomyelinase activity [10]. Because of the sphingomyelin-mediated sequestration of cholesterol [14] and the dependence ALN has on cholesterol [5,7], we hypothesized that exposing human erythrocytes to PLD would increase the amount of accessible cholesterol, measurable by an increase in ALN-mediated hemolysis, in response to longer exposure to PLD. Sub-hemolytic concentrations of ALN were added to human erythrocytes for 30 min at 22 °C, followed by the addition of sub-hemolytic concentrations of PLD. In this order, ALN followed by PLD, no observable increase in hemolysis was detected (Figure 3A).

However, when the reciprocal experiment was carried out, incubating human erythrocytes first with PLD followed by ALN, ALN-mediated hemolysis greatly increased in response to higher concentrations (Figure 3B) or longer incubations of PLD (Figure 3C). Together, these experiments demonstrate that PLD promotes ALN-mediated hemolysis in an order-dependent fashion.

### 2.4. An Increase in ALN-Mediated Hemolysis Is Not Dependent upon the Generation of Cyclic-Ceramide Phosphate

**Figure 4:**
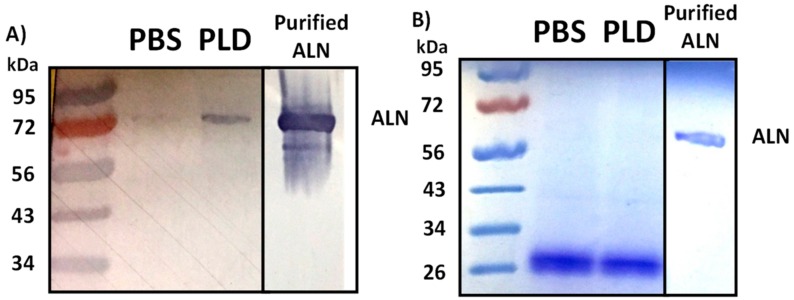
Analysis of ALN-binding in response to PLD treatment of whole human blood. (**A**) Whole human blood was treated with PBS only or PBS containing 1 µg/mL PLD for 60 min at 37 °C with gentle agitation. PLD-treated samples were washed with PBS, chilled on ice, and equal parts blood and ice-cold ALN were incubated on ice with gentle agitation for 60 min. Samples were washed three times with 1× PBS, suspended in loading buffer, and examined via Western blot using an anti-ALN antibody as described in Materials and Methods. Lane 1: protein ladder, Lane 2: RBCs treated with 1× PBS, followed by ALN, Lane 3: RBCs treated with 1 μg/mL PLD, followed by ALN, Lane 4: Purified His-ALN only. (**B**) Equal loading was ensured using Coomassie staining to determine an identical amount of erythrocytes were present for each sample.

In order to evaluate if the increase in ALN-mediated hemolysis is dependent upon eliminating the ability of SM to sequester cholesterol or is dependent on the generation of the unique cyclic ceramide derivative by PLD, we purified a recombinant form of sphingomyelinase D (SPH) produced by *Bacillus anthracis* [15]. SPH is well-characterized biochemically and converts sphingomyelin to phosphocholine and ceramide; no cyclic products are generated [15]. Similar to the experiments done in Figure 4, we incubated human erythrocytes with increasing concentrations of SPH followed by the addition of sub-hemolytic concentrations of ALN. Incubating erythrocytes with SPH led to an increase in ALN-mediated hemolysis in a concentration-dependent manner (Figure 3D). 

### 2.5. Treatment of Human Erythrocytes with PLD Subsequently Promotes ALN-Membrane Binding

CDCs have long been known to follow a defined process in forming pores, starting with monomer binding to the host membrane [6,7]. Two separate regions within Domain 4 of CDCs are critical in the recognition and interaction with cholesterol: a tryptophan (Trp)-rich region known as the undecapeptide [16] and a threonine-leucine (Thr-Leu) amino acid motif [17]. The primary structure of ALN contains both an undecapeptide region (AAs 525–535) and a Thr-Leu motif (AAs 557–558) and its pore-forming activity has previously been shown to be subject to cholesterol availability within the target membrane [5]. Furthermore, point mutations within either of the motifs negatively impacts ALN-mediated hemolysis, which suggests the ability of ALN to recognize and interact with cholesterol is necessary for hemolysis (data not shown). Based on these observations, we hypothesized that by increasing the pool of ALN-accessible cholesterol, the enzymatic activity of PLD would increase the ability of ALN to bind to treated erythrocyte membranes. Erythrocytes were incubated with PLD or PBS alone, washed, and subsequently chilled and incubated with ALN to allow the CDC to bind to the erythrocyte but prevent subsequent oligomerization and pore-formation. The amount of ALN able to bind to erythrocytes was examined via Western blot. We observed a substantial increase in ALN bound to erythrocytes treated with PLD compared to erythrocytes treated with PBS alone (Figure 4). Taken in context with the data previously discussed, *A. haemolyticum* PLD promotes ALN-mediated hemolysis by promoting the initial binding step of the ALN pore-formation process.

### 2.6. Individual Point Mutations within PLD Decreases Sphingomyelinase Activity

**Figure 5:**
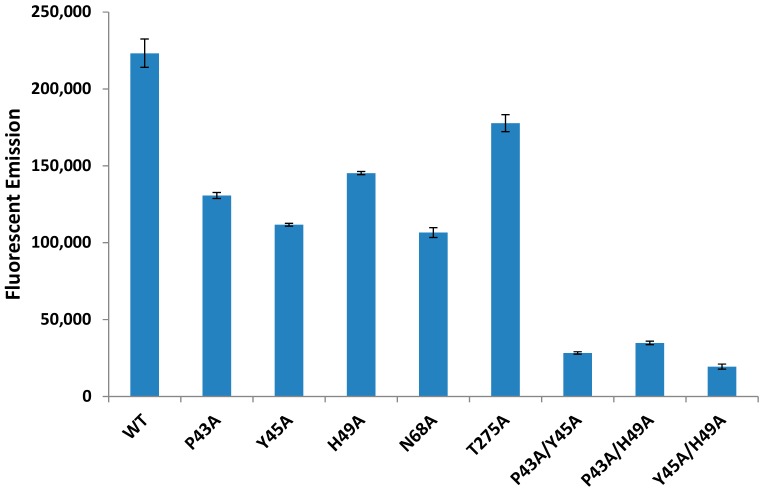
Mutating specific amino acids in PLD is sufficient to diminish the sphingomyelinase activity of PLD. *E. coli* containing a mutated pTrcHis*pld* plasmid were grown overnight, lysed via sonication, and sphingomyelinase activity was measured using the Amplex Red Sphingomyelinase Assay kit. Bacteria containing the plasmid with one mutation possessed sphingomyelinase activity between 50 and 80% that of WT PLD while bacteria containing a plasmid with two mutations had between 8 and 15% WT PLD sphingomyelinase activity. Data is representative of three separate experiments and values represent the mean with error bars showing standard deviation. The fluorescent emission of a buffer only negative control was subtracted from each of the treatments above.

Alignments of homologous bacterial and fungal phospholipase D enzymes revealed a number of amino acids that appear to be conserved (Supplementary Materials Figure S1). We identified several amino acids within PLD as candidates for mutagenesis (P43, Y45, H49, N68, H272, and T275). Individual amino acids were mutated to an alanine as described in the Materials and Methods section and the sphingomyelinase activity of each PLD variant was evaluated. Individual point mutations were sufficient to decrease sphingomyelinase activity to 47.7–79.6% of WT PLD levels (Figure 5). 

We identified the most effective single mutations and created double mutations (P43A/Y45A, P43A/H49A, and Y45A/H49A) and purified these PLD variants. All sets of double point mutations decrease sphingomyelinase activity levels to 8–15% of WT PLD levels (Figure 5). The presence and stability of PLD variants was verified by SDS-PAGE gels stained with Coomassie Brilliant Blue staining (Data not shown).

### 2.7. Sphingomyelinase-Deficient PLD Fails to Promote ALN-Mediated Hemolysis to the Same Extent as WT-PLD

**Figure 6:**
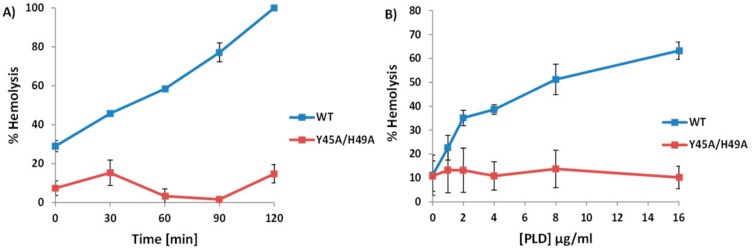
Sphingomyelinase-deficient PLD fails to promote ALN-mediated hemolysis. (**A**) 2 µg/mL WT or Y45A/H49A PLD was incubated with 2% human erythrocytes for between 0 and 120 min followed by the addition of 20 ng/mL ALN for an additional 30 min at 37 °C. (**B**) Varying concentrations of WT or Y45A/H49A PLD were incubated with 2% human erythrocytes for 30 min followed by the addition of 20 ng/mL ALN for an additional 30 min at 37 °C. In both cases, WT PLD increased ALN-mediated hemolysis while mutated PLD failed to promote ALN-mediated hemolysis. Each experiment was conducted three times in triplicate. Representative experiments are presented here with standard deviation represented by error bars.

We hypothesized that the enzymatic activity of PLD was necessary to promote ALN-mediated hemolysis. Therefore, experiments outlined in Figure 3 were repeated but included the SMase-deficient PLD, with SMase activity below 10% of WT PLD SMase activity, Y45A/H49A (Figure 6A). 

Human erythrocytes were incubated with identical concentrations of WT or Y45A/H49A PLD followed by sub-hemolytic concentrations of ALN. While the WT PLD was able to promote ALN-mediated hemolysis, the mutated PLD failed to promote ALN-mediated hemolysis in neither time- (Figure 6A) nor concentration-dependent manners (Figure 6B). This observation suggests that the enzymatic activity of PLD is necessary to promote the pore-forming activity of ALN. 

### 2.8. The PLD-ALN Synergistic Relationship Also Exists in a Physiologically-Relevant Cell Line

**Figure 7:**
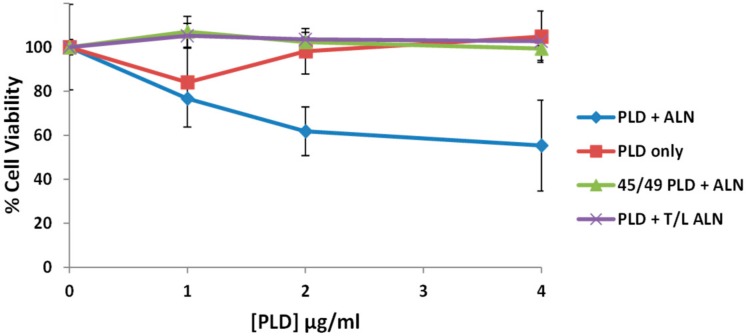
The PLD-ALN synergism also occurs in Detroit 562 cells. Detroit 562 cells were cultured and seeded into 96-well plates and allowed to adhere overnight. Cells were then washed with PBS and treated with medium containing 0–4 μg/mL WT or SMase-PLD and incubated for 1 h at 37 °C. Cells were then washed and treated with medium containing varying concentrations of WT or T/L ALN for 30 min at 37 °C. After the incubation, all wells were treated with the MTS reagent to measure cell viability. After a 60 min incubation at 37 °C, the OD_490_ was measured and used to calculate the % cell viability of cells treated with ALN alone or a combination of PLD followed by ALN. No observable decrease in cell viability was detectable when ALN only was added to the cells, but a 45% decrease in cell viability was observed when both PLD and ALN were added into the system. Each experiment was conducted three times with a representative experiment presented above. Error bars represent standard deviation.

While hemolysis assays are an effective tool to measure CDC pore-formation, it is very unlikely that erythrocytes are a biologically relevant target for ALN or PLD and there is no literature suggesting the *A. haemolyticum* lifecycle includes erythrocytes. Therefore, we opted to test our model in a more physiologically relevant cell line: the immortalized Detroit 562 pharyngeal epithelial cell line. Cells were seeded in a 96-well plate overnight, washed, treated with medium containing various amounts of WT or Y45A/H49A PLD followed by WT ALN or a T/L mutated ALN which fails to recognize cholesterol and has no hemolytic activity at concentrations of 100 μg/mL or below (Data not shown). The MTS reagent was added to each well 30 min after the proteins and cells had incubated to measure cell viability (Figure 7). In this assay, ALN alone was insufficient to affect cell viability but was able to decrease cell viability when added to cells pre-treated with PLD, supporting our model in a physiologically relevant cell line. In addition, when either mutated ALN or mutated PLD were substituted for their WT counterparts, no observable decrease in cell viability was observed.

## 3. Discussion

*A. haemolyticum* is a human bacterial pathogen that has received very little attention. Since its discovery in 1946 [18], two proteins of *A. haemolyticum* have been identified and partially characterized: A sphingomyelin-specific PLD [8,10] and ALN [5], a member of the CDC family of pore-forming toxins. CDCs constitute a family of pore-forming toxins known to play critical roles in various aspects of bacterial lifecycles and pathogenesis [7]. A hallmark of the 50+ identified CDCs is that they appear to follow the same pore-forming choreography, but investigation of CDCs individually as well as within the context of an entire bacterial repertoire reveal characteristics unique to each CDC. Therefore, it is important to study the properties of each CDC individually as well as in the context with other bacterial factors to fully understand this unique family of pore-forming toxins and their function in bacterial lifecycles and human pathogenesis. We set forth to further our understanding of the pore-forming ability of ALN in the context of the sphingomyelinase-specific PLD from *A. haemolyticum*.

Like the majority of CDCs, ALN requires membrane cholesterol to mediate pore-formation [5]. The ability of ALN to interact with cholesterol can be impeded by umbrella-like covering over cholesterol from its interactions with sphingomyelin or phosphatidylcholine [11]. Many of the CDC-producing bacteria, including *A. haemolyticum*, produce and secrete enzymes capable of removing the head group of sphingomyelin [10], eliminating the sphingomyelin-cholesterol interaction and increasing cholesterol accessibility [11,12]. Using purified ALN and PLD, we provide evidence that the sphingomyelinase activity of PLD promotes ALN-mediated hemolysis in both time- and concentration-dependent manners. Overall, data presented in this study suggests *A. haemolyticum* PLD and ALN synergistically work together to form pores within a host membrane.

A key next step will be to investigate how the synergistic relationship among PLD and ALN affects interactions between *A. haemolyticum* and host epithelial cells. PLD induces the formation of lipid rafts [8], cholesterol-rich platforms that have long been hypothesized as sites of interaction between bacteria and host cells [19]. Loss of PLD was previously reported to significantly decrease *A. haemolyticum* adherence to and invasion of host cells in vitro [8]; however, no mechanism has been described. It has long been accepted that disturbances within plasma membranes, specifically pore-formation by CDCs, induces internalization of damaged plasma membrane [20]. It is tempting to speculate that a similar process may be hijacked by *A. haemolyticum* to induce its uptake into non-phagocytic cells. 

In addition, two studies have demonstrated that *A. haemolyticum* initially resides within an unidentified vesicle during its lifecycle before entering into the host cytoplasm and inducing necrosis [8,21]. While the mechanism by which *A. haemolyticum* is initially internalized into the vesicle is unknown, *A. haemolyticum* appears to mediate its escape into the host cytoplasm in a PLD-dependent manner [8]. The lumen of an early endosome is known to be particularly enriched in sphingomyelin [22], suggesting the sphingomyelinase activity of PLD, in addition to the ALN pore-formation activity, may play an integral role during the intracellular phase of the *A. haemolyticum* lifecycle. Whether or not PLD and ALN work in tandem to promote the internalization of *A. haemolyticum* has not yet been reported, but remains an area of ongoing investigation in our laboratory.

In addition to *A. haemolyticum*, many CDC-producing bacteria are also known to produce and secrete phospholipases capable of acting on a variety of phospholipids including sphingomyelin, phosphatidylcholine, or phosphatidylinositol [15,23,24,25]. This study focused on a lipase previously shown to act only on sphingomyelin and its ability to promote the pore-forming activity of Arcanolysin. However, many CDC-producing bacteria including *Clostridium perfringens*, *Bacillus anthracis*, *Bacillus cereus*, and *Listeria monocytogenes* produce and secrete two separate phospholipases: a sphingomyelinase-specific lipase and a phosphatidylcholine-specific lipase [15,23,24]. In this study we also noted that the *B. anthracis* SPH enzyme also promotes ALN-mediated hemolysis, suggesting that the increase in the hemolytic activity of ALN is not increased due to an increase in a particular lipid (choline in the case of SPH or cyclic-ceramide phosphate in the case of PLD) but rather due to a decrease in cholesterol sequestering by sphingomyelin. Interestingly, SPH and PLD share virtually no amino acid homology, which could suggest each bacterium acquired their respective lipase and CDC by independent means. While our work demonstrates the effect sphingomyelinase activity has on ALN-mediated hemolysis, it is unknown how altering other phospholipids would affect ALN-mediated hemolysis or the hemolytic activity of other members of the cholesterol-dependent cytolysin family. Of particular interest would be phosphatidylcholine as it is also known to transiently interact with and shield cholesterol from extracellular factors (9).

Our analysis of PLD did seemingly contradict with the initial characterization of the purified enzyme. Recombinant PLD was initially reported to have no hemolytic activity [8]. However, we observed hemolysis when 20–40 µg/mL PLD was incubated with human erythrocytes for 30 min. The previous publication did not note the maximum concentrations used to evaluate the hemolytic activity of PLD and therefore may not have approached the minimum concentration of PLD or time period necessary to disrupt the membrane and lyse cells. The other question regarding the hemolytic activity of PLD is its mechanism of lysis. PLD is known to remove the choline head of lysophosphatidylcholine and sphingomyelin, two activities which would not be sufficient to lyse a membrane. However, there is a growing body of literature which suggests that altering the head groups of phospholipids, in particular sphingomyelin, can induce dramatic changes in membrane curvature and induce internalization of portions of a membrane [26]. It is easy to envision a point in which a critical mass of sphingomyelin within a plasma membrane has been converted into its cyclic-ceramide derivative by PLD and can no longer support the natural curvature of an intact membrane, causing lysis of the cell.

Overall, this study emphasizes the need to study the biochemistry of CDCs on an individual basis as well as in the context of other bacterial factors. Herein, we demonstrated the existence of a lipase-CDC synergistic relationship, but it is tempting to speculate on the possibility that other bacterial enzymes, such as proteases or amylases, affect membrane composition and influence the ability of CDCs to interact with and form pores within a host membrane. Understanding the interactions among multiple virulence factors and host cells may be essential to unraveling the complex and dynamic interplay between hosts and pathogens.

## 4. Materials and Methods 

### 4.1. Bacteria and Growth Conditions

All *A. haemolyticum* strains used originated from the ATCC9345 strain. *A. haemolyticum* strains were grown at 37 °C with 5% CO_2_ on campylobacter agar plates supplemented with 5% human blood from normal, healthy donors. *A. haemolyticum* strains were grown in Todd-Hewitt broth supplemented with 5% fetal bovine serum at 37 °C with 5% CO_2_ with shaking. A total of 25 µg/mL kanamycin (Kn), 5 µg/mL chloramphenical (Cm), or 10 µg/mL erythromycin (Er) were added to broth or agar when appropriate. 

### 4.2. Construction of A. haemolyticum Mutants and Complementing Plasmids

The Δ*pld* and Δ*pld*:*pld*+ strains were generously provided by the Stephen Billington and Helen Jost laboratories (University of Arizona, Tucson, AZ, USA). The Δ*aln A. haemolyticum* strain was constructed by PCR amplifying *aln* from the ATCC9345 strain of *A. haemolyticum* using the forward primer (5′-CGGGATCCGTCAAGTTATGCCGGGAATG-3′) and reverse primer (5′-TCCGAGCTCGTTCTTGAACCAAGG-3′). The product was digested using SacI and BamHI and inserted into the intermediate vector pCRTopo creating pCRTopo*aln*. The *aln* gene was interrupted using BamHI and EcoRI enzymes with a 1.4-kb Kn resistance gene from pBS-Kan [27] digested with EcoRI. This resulted in an internal *aln* deletion of base pairs 656 to 763. The resultant plasmid, pBS-*aln*::Kan, was electoporated into the ATCC9345 strain of *A. haemolyticum* and transformants were screened by PCR and Western blot to verify the presence of the interrupted *aln* gene and absence of ALN protein, respectively (data not shown). A complementing *aln* plasmid, pJGS182*aln*, was constructed by digesting both pCRTopo*aln* with BamHI and SacI to release a ~2.0 kb product which included the full *aln* gene and promoter. The *aln* gene and promoter were then inserted into a pJGS182 vector digested with BamHI and SacI. pJGS182 is a pNG2 and pEP2 derivative containing *ermX* and an improved multi-cleaving site [28,29] The pJGS182*aln* plasmid was electroporated into the Δ*aln A. haemolyticum* strain, conferring both ALN production and erythromycin and kanamycin resistance.

### 4.3. DNA Techniques and Sequence Analysis

Plasmid DNA extraction and transformation using *E. coli* DH5αMCR were carried out by standard methods. DNA restriction, ligation and agarose gel electrophoresis, and PCR DNA amplification were carried out as previously described [5,8]. 

### 4.4. Purification of Recombinant His-PLD, His-ALN, His-SPH, and His-RocF

Starting cultures of Top10F’ *E. coli* containing either pTrcHis*aln*, pTrcHis*pld*, plasmids containing a mutated *aln* or *pld*, pTrcHisB, pET-15b-*antB*, or pQE-*rocFb* were grown overnight in LB broth with 200 µg/mL ampicillin. Overnight cultures were diluted 1:100 in a total of 1 L of LB broth with ampicillin and grown at 37 °C with shaking at 225 rpm until the OD600 nm reached 0.6. Isopropyl β-d-1-thiogalactopyranoside (IPTG) was added to the culture to reach a final concentration of 2.5 mM and incubated for an addition 4 h. Cells were centrifuged at 6700× *g* for 15 min at 4 °C. Supernatants were removed and bacterial pellets were resuspended in 12.5 mL wash/lysis buffer (50 mM NaH_2_PO_4_, 300 mM NaCl, 15 mM imidazole, pH 8.0) and lysed using a French press twice at 16,000 psi. The remainder of the protocol was completed at 4 °C. Pressed lysates were centrifuged at 9600× *g* and the supernatant was transferred to a fresh 50 mL conical tube with Ni-NTA resin (Qiagen, Carlsbad, CA, USA) at a 2:1 ratio. The supernatant/resin slurry was gently mixed end-over-end in the cold room overnight followed by transfer to sterile 5 mL polypropylene columns. Columns were washed 10 times with ~6 mL wash/lysis buffer and His-tagged proteins were collected in 1 mL aliquots with elution buffer (50 mM NaH_2_PO_4_, 300 mM NaCl, 250 mM imidazole, pH 8.0). Fractions were analyzed using SDS-PAGE gels and Coomassie Blue stain to evaluate which aliquots contained protein. Elutions containing samples were combined and dialyzed in 1× PBS using Slide-A-Lyzer cassettes (ThermoFisher Scientific, Waltham, MA, USA) overnight and protein concentrations were calculated using a standard bicinchoninic acid assay (Pierce, Rockford, IL, USA).

### 4.5. Hemolytic Assays 

Hemolytic activities of His-ALN and His-PLD were first determined by serially diluting each protein in 1× PBS and incubating each dilution with human erythrocytes suspended in 1× PBS. A total of 100 µL of protein was incubated with 100 µL 2% washed erythrocytes at 37 °C for 30 min in 96-well plates. Following the incubation, plates were centrifuged at 15 °C for 10 min at 4200× *g* to pellet unlysed erythrocytes. Supernatants were transferred to a fresh, flat-bottom 96-well plate and OD_415_ nm readings were recorded using a FLUOstar Omega plate reader. 

### 4.6. ALN-Binding Assays

Whole, human blood was treated with 1 μg/mL PLD for 60 min at 37 °C for 60 min with gentle agitation, washed with 1× PBS three times, and chilled on ice for 15 min. Ice-cold, purified ALN was then added to the chilled erythrocytes for 60 min at 4 °C with gentle agitation to allow ALN to bind to the erythrocytes but slow the lateral diffusion and oligomerization of CDCs [30]. Samples were centrifuged at 4 °C and washed with ice-cold PBS to prevent lysis, suspended in loading buffer and run in a 10% SDS-PAGE gel and transferred onto Immobilon PVDF Membrane (Millipore, Burlington, MA, USA) or stained with Coomassie as a loading control. Blots were blocked with 5% non-fat dry milk dissolved in Tris-buffered saline and Tween 20 (TBST; 0.5% Tween 20) for 2 h at room temperature (RT). Washes between blocking, primary and secondary antibodies were performed three times for 10 min. Blots were incubated with with a rabbit Anti-His-ALN primary antibody (adsorbed against *E. coli* containing pTrcHisB) (1:1000) (ProMab Biotechnologies, Inc., Richmond, CA, USA) and an Anti-Rabbit IgG (whole molecule) Peroxidase conjugate secondary antibody (1:10,000) (Sigma, St. Louis, MO, USA). Blots were developed with nitroblue tetrazolium/indoxyl phosphate (NBT/IP) (100 µg/mL and 1 mg/mL, respectively) in glycine buffer (0.1 M glycine, 1 mM ZnCl_2_, 0.1% NaN_3_) at 37 °C for 30 min.

### 4.7. Sphingomyelinase Assays

Top10F’ *E. coli* containing the WT or mutated pTrcHis*pld* plasmids were grown in LB broth overnight at 37 °C. Aliquots of bacteria were centrifuged for 10 min at 9600× *g*. Bacterial pellets were suspended in lysis buffer and sonicated for 30 s at 40% amplitude a total of three times using a Sonic Dismembrator 500 (Fisher Scientific, Hampton, NH, USA). Lysates were then incubated with a master mix from an Amplex Red Sphingomyelinase Assay kit (Invitrogen, Eugene, OR, USA) (200 µM Amplex Red Reagent, 1 U horseradish peroxidase/mL, 4 U alkaline phosphatase/mL, 0.1 U choline oxidase/mL, and 0.25 mM sphingomyelin in 0.1 M Tris-HCl-10 mM MgCl_2_). The assay was allowed to incubate at 37 °C for 30 min protected from light. Fluorescent emissions were measured using a FLUOstar Omega plate reader with excitation at 544 nm and detection at 590 nm.

### 4.8. Mutagenesis of Pld or Aln

Point mutations were engineered into pTrcHis*pld* or pTrcHis*aln* using the QuikChange XL Site-Directed Mutagenesis kit (Agilent Technologies, Cedar Creek, TX, USA). Briefly, PCR products originating from pTrcHis*pld* or pTrcHis*aln* and corresponding primers (Table 1) were incubated with DpnI and heat shocked into Top10F’ *E. coli* and grown overnight on LB plates supplemented with ampicillin. Transformants were selected and SMase activity levels of *E. coli* lysates were measured using the Amplex Red Sphingomyelinase Assay kit. Plasmids from transformants with decreased SMase levels were sequenced (Arizona State University DNA Sequencing Lab, Tempe, AZ, USA) to confirm the presence of the mutation.

### 4.9. Cell Viability Assays

Detroit 562 cells were grown in F-12 Ham’s Medium supplemented with 10% FBS. Approximately 1 × 10^4^ cells were seeded in a Costar 96-well flat bottom plate and allowed to adhere overnight. Cells were then washed with with 1× PBS a total of three times and the medium was replaced with F-12 Ham’s Medium alone (lacking FBS) containing various concentrations of PLD and incubated for 1 h at 37 °C. Cells were then washed and the medium was replaced with F-12 Ham’s Medium (no FBS) containing various concentrations of ALN. Cells were allowed to incubate for 30 min at 37 °C, at which point 20 μL of MTS Reagent (Biovision Incorporated, Milpitas, CA, USA) was added to each well. After the MTS Reagent had been added to the cells for 60 min, OD_490_ nm readings were taken and, with a medium-only negative control and Trition X-100 positive control, used to calculate cell viability. 

## Figures and Tables

**Table 1:** List of primers used in this study.

Primer Name	Sequence (5′-3′)
DM1117 (T557A_Forward)	CGCGAGATCATCCTTCGCGGTACTGCCTTACGGCCTTGGTTC
DM1118 (T557A_Reverse)	GAACCAAGGCCGTAAGGCAGTACCGCGAAGGATGATCTCGCG
DM1121 (L558A_Forward)	CGCGAGATCATCCTTCGCGGTACTACCGCACGGCCTTGGTTC
DM1122 (L558A_Reverse)	GAACCAAGGCCGTGCGGTAGTACCGCGAAGGATGATCTCGCG
DM1125 (TL557_58AA_Forward)	CGCGAGATCATCCTTCGCGGTACTGCGGCACGGCCTTGGTTC
DM1126 (TL557_58AA_Reverse)	GAACCAAGGCCGTGCCGCAGTACCGCGAAGGATGATCTCGCG
DM1162 (P43A_Forward)	ACCAACCACTGGTAACCGTGCCGTCTATGCCATTGCGCACC
DM1163 (P43A_Reverse)	GGTGCGCAATGGCATAGACGGCACGGTTACCAGTGGTTGGT
DM1164 (Y45A_Forward)	AGAACACGGTGCGCAATGGCGGCGACTGGACGGTTACCAGTGG
DM1165 (Y45A_Reverse)	CCACTGGTAACCGTCCAGTCGCCGCCATTGCGCACCGTGTTCT
DM1166(H49A_Forward)	TTTGCTTCGTCAGAACACGGGCCGCAATGGCATAGACTGGAC
DM1167(H49A_Reverse)	GTCCAGTCTATGCCATTGCGGCCCGTGTTCTGACGAAGCAAA
DM1168(N68A_Forward)	AGTAAAATCAATTTCCAGAGCGGCCGCGCCAATTTTGATTGCGTC
DM1169(N68A_Reverse)	GACGCAATCAAAATTGGCGCGGCCGCTCTGGAAATTGATTTTACT
DM1170(H272A_Forward)	AATGTCTTTGTTGGTTGCCATGGCGTGAGTACCTTGATGTGCATC
DM1171(H272A_Reverse)	GGATGCACATCAAGGTACTCACGCCATGGCAACCAACAAAGACAT
DM1172(T275A_Forward)	ACGGAATGTCTTTGTTGGCTGCCATGTGGTGAGTACCTTG
DM1173(T275A_Reverse)	CAAGGTACTCACCACATGGCAGCCAACAAAGACATTCCGT
DM1174 (P43A,Y45A_F)	CGGTGCGCAATGGCGGCGACGGCACGGTTACCAGTGGTTGGTTGTTCTT
DM1175 (P43A,Y45A_R)	AAGAACAACCAACCACTGGTAACCGTGCCGTCGCCGCCATTGCGCACCG
DM1176 (P43A,H49A_F)	CTTTGCTTCGTCAGAACACGGGCCGCAATGGCATAGACGGCACGG
DM1177 (P43A,H49A_R)	CCGTGCCGTCTATGCCATTGCGGCCCGTGTTCTGACGAAGCAAAG
DM1178 (Y45A,H49A_F)	GCTTCGTCAGAACACGGGCCGCAATGGCGGCGACTGGACGGTTACCAG
DM1179 (Y45A,H49A_R)	CTGGTAACCGTCCAGTCGCCGCCATTGCGGCCCGTGTTCTGACGAAGC

## Supplementary Materials

The following are available online at http://www.mdpi.com/2072-6651/10/6/213/s1, Figure S1: Sequence alignment of various PLD amino acid sequences.
