# Supplementary material for: Arcanobacterium haemolyticum Phospholipase D Enzymatic Activity Promotes the Hemolytic Activity of the Cholesterol-Dependent Cytolysin Arcanolysin
Source: Toxins (Basel). 2018 May 23;10(6):213. doi: 10.3390/toxins10060213 (PMC6024514; doi:10.3390/toxins10060213)
Supplement: Supplementary file 1 [file toxins-10-00213-s001.pdf]

Toxins **2018**, *10*, 213; doi:10.3390/toxins10060213

Supplementary Materials: *Arcanobacterium haemolyticum* Phospholipase D Enzymatic Activity Promotes the Hemolytic Activity of the Cholesterol-Dependent Cytolysin Arcanolysin

Patrick S. Gellings and David J. McGee

```
PLD_Dcongolensis     MSSRAMKVSIAAVVAAGSFLAASPVHAAPAT-PSNPASQRPVYAIAHRVLTVKGVDDAVK    59
PLD_Ahaemolyticum    MKTRK-KIALALSLLTGFMLPIGSAAAAPLAQEQPTTGNRPVYAIAHRVLTKQSVDDAIK    59
PLD_Cpseudotb        --MRE-KVVLFLSIIMAIMLPVGNAAAAPVVHNPASTANRPVYAIAHRVLTTQGVDDAVA    57
PLD_Culcerans        --MKK-KIVLFLSIIMGILLPVGNAVATPVSHDAASTGNRPVYAIAHRVLTTQGVDDAVA    57
                        :  *: :   :  . :*  . . *:*       :.:************ :.****:

PLD_Dcongolensis     LGANAIEIDLTARKSGWSADHDGLPTSAGDSAETMFKHIAAKKKAGSNISFIWLDIKNPD    119
PLD_Ahaemolyticum    IGANALEIDFTAWRRGWWADHDGLPTSAGDTAEDILKYIAQKRREGNNITFVWFDIKNPD    119
PLD_Cpseudotb        IGANALEIDFTAWGRGWWADHDGIPTSAGATAEEIFKHIADKRKQGANITFTWLDIKNPD    117
PLD_Culcerans        IGANALEIDFTAWRGGWWADHDGIPTSAGATAEAIFKHIAEKRRQGANITFTWLDIKNPD    117
                     :****:***:**   ** *****:***** :** ::*:** *:: * **:* *:******

PLD_Dcongolensis     YCKSGEAGSKCSIDHLRDLARNTFEKEGVRALYGFYKTAGAAAWNTITHDLNDKEAVALS    179
PLD_Ahaemolyticum    YCKD--QNSVCSITKLRDLARQTIEQEGVRALFGFYKTVGGVGWNTIANNLNDKEAVALS    177
PLD_Cpseudotb        YCRD--ARSVCSINALRDLARKYLEPAGVRVLYGFYKTVGGPAWKTITADLRDGEAVALS    175
PLD_Culcerans        YCTD--PDSVCSINALRDLARKYLEPAGVRVLYGFYKTVGGPGWKTITSDLRDKEAVALS    175
                     ** .    * ***  ******: :*  ***.*:*****.*. .*:**: :*.* ******

PLD_Dcongolensis     GPTETVLSDYNKSKKPVPVNKRIADYGFFNLRLGFGSCTGTAKKTCDQLRLSNEARDKGK    239
PLD_Ahaemolyticum    GRKDDIMKDFKQYENKIKPQQRVADNGYYNLSYGFGGCYRDENQTCDQLRLAGEERKKGN    237
PLD_Cpseudotb        GPAQDVLNDFARSENKILTKQKIADYGYYNINQGFGNCYGTWNRTCDQLRKSSEARDQGK    235
PLD_Culcerans        GPTRDVLNDFAKAGDKILTKQKIADYGYYDINQGFGNCYGDGNKTCDQLRKSSEARDQGQ    235
                     *    ::.*: :  . :  ::::** *::::  ***.*    ::****** :.* *.:*:

PLD_Dcongolensis     LGKVFGWTVTGKQKDIVSDLLGKAHVDGLIAGFKATNFYEHPDSKASLKNIHDWVAAHPQ    299
PLD_Ahaemolyticum    LGKTFGWTVSTGQEYLAADLLNKAEVDGMIFGFKTTYFYDHADTRNAFAGIKNWVDAHQG    297
PLD_Cpseudotb        LGKTFGWTIATGQDARVNDLLGKANVDGLIFGFKITHFYRHADTENSFKAIKRWVDKHSA    295
PLD_Culcerans        LGKTFGWTITTGQDDRVNDLLGKAHVDGMIFGFKVTHFYRHADTENSFKAIKTWVDKHSD    295
                     ***.****::  *.  . ***.**.***:* *** * ** * *:. ::  *: **  *

PLD_Dcongolensis     THRMATANDKPW                 311
PLD_Ahaemolyticum    THHMATNKDIPW                 309
PLD_Cpseudotb        THHLATVADNPW                 307
PLD_Culcerans        THHLATAADNPW                 307
                     **::**  * **
```

Figure S1. Sequence alignment of various PLD amino acid sequences. PLD amino acid sequences from *Arcanobacterium haemolyticum* (WT_041640381.1), *Corynebacterium pseudotuberculosis* (WP_013240889.1), *Corynebacterium ulcerans* (WP_014525098.1) and *Dermatophilus congolensis* (WP_028327396.1) were aligned using Clustal Omega analysis. Amino acids highlighted in yellow were selected as candidates for mutagenesis to alanine.
